# Strain-Mediated Bending of InP Nanowires through the Growth of an Asymmetric InAs Shell

**DOI:** 10.3390/nano9091327

**Published:** 2019-09-16

**Authors:** Ya’akov Greenberg, Alexander Kelrich, Shimon Cohen, Sohini Kar-Narayan, Dan Ritter, Yonatan Calahorra

**Affiliations:** 1 Department of Electrical Engineering, Technion, Haifa 32000, Israel; 2 Department of Materials Science and Metallurgy, University of Cambridge, Cambridge CB3 0FS, UK

**Keywords:** nanowires, strain engineering, simulation, core–shell, asymmetry, bending

## Abstract

Controlling nanomaterial shape beyond its basic dimensionality is a concurrent challenge tackled by several growth and processing avenues. One of these is strain engineering of nanowires, implemented through the growth of asymmetrical heterostructures. Here, we report metal–organic molecular beam epitaxy of bent InP/InAs core/shell nanowires brought by precursor flow directionality in the growth chamber. We observe the increase of bending with decreased core diameter. We further analyze the composition of a single nanowire and show through supporting finite element simulations that strain accommodation following the lattice mismatch between InP and InAs dominates nanowire bending. The simulations show the interplay between material composition, shell thickness, and tapering in determining the bending. The simulation results are in good agreement with the experimental bending curvature, reproducing the radius of 4.3 µm (±10%), for the 2.3 µm long nanowire. The InP core of the bent heterostructure was found to be compressed at about 2%. This report provides evidence of shape control and strain engineering in nanostructures, specifically through the exchange of group-V materials in III–V nanowire growth.

## 1. Introduction

One-dimensional nanostructures have been studied in the past decades for efficient applications in electronics, optics [1,2], electromechanics, energy [3,4], and more. Advanced control over nanomaterial shape provides a useful route for improving its electronic or optical properties. This has been manifested in semiconductor nanowires (NWs) through several routes: (i) the growth of NWs with asymmetric cross section [5,6], where the NW cross-sectional shape was found to direct the light emission from the NW [5]; (ii) growth direction switching [7]; (iii) guided planar growth [8,9], opening up possibilities for straightforward electronic and optoelectronic NW integration; (iv) formation of unintentional nanostructures, such as quantum wires on NW facets, introducing unique optical and charge transfer properties [10,11]; and (v) growth of nanoflags [12], flakes/membranes [13,14,15], and selective area nanostructures [16,17]. These structures have been shown to influence, for example, direct, optical emission from nanostructures [12,16,18]. 

Another avenue recently reported is the growth of bent NWs, through realizing asymmetry in the formation of a core–shell structure, and the effect of strain on the NW optical properties [19]. Despite several previous observations of unintentionally bent NWs [20,21,22,23,24], only the recent work by Lewis et al. showed deliberate bent growth and its analysis [19]. Controllable strain-mediated bending requires directionality; therefore, bending in vapor-phase epitaxy systems, where a (relatively) uniform gaseous phase surrounds the NWs, is random [21,23,25]. Similarly, bent NWs grown in molecular beam epitaxy (MBE) systems where substrate is rotated show random directionality [24,26]. The role of geometry, chemical composition, and the interplay between strain relaxation and surface energy in the growth of bent NWs have been examined [25,27]. Alternatively, molecular beam epitaxy system without rotation are directional and induce an inherent asymmetry directly related to bending [19,20]. Strain modifies the fundamental properties of semiconductor, such as the bang gap and effective mass, and can change electronic, optical, and mechanical properties [28,29,30,31]. Here we report the induced bending of InP NWs through the growth of an asymmetric InAs shell. The effects of NW separation (pitch) and diameter on bending are shown. In addition, a single NW case study is presented: the bending is quantified and correlated to the chemical composition of the NW. We further explore the effects of geometry and composition on the strain relaxation through finite element simulation and find high correspondence to the experimental NW curvature by accurately accounting for NW shape, tapering in particular. 

## 2. Experimental Details

**Figure 1:**
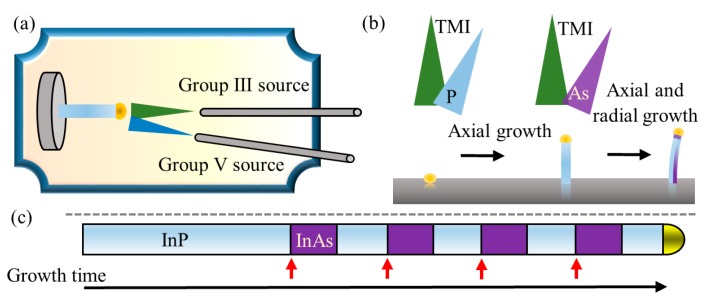
(**a**) Schematic of the metal–organic molecular beam epitaxy (MOMBE) growth chamber showing the vertical tube used to introduce group-III growth species to the system and the 30° inclined tube used for group-V species. (**b**) Schematic of the growth procedure of InP/InAs nanowire (NW) heterostructures and the resulting bent NW. (**c**) Schematic of the growth sequence illustrating the nominal NW form, where InAs (red arrows) was introduced four times following the growth of an InP NW. Note that the actual NW shape is significantly different.

Nanowire growth procedure followed previous reports [32,33]. In short, InP <111> B (phosphorous terminated) substrates (Semiconductor Wafer Inc., Hsinchu, Taiwan) were covered with a 150 Å silicon nitride by plasma-enhanced chemical vapor deposition (PECVD, Plasma-Therm 790, FL, USA) and patterned by hydrofluoric acid (HF) etching after following electron-beam lithography (Raith, EBPG5200, Dortmund, Germany). Afterward, gold evaporation of 50 Å thick layer was carried out for lift-off to obtain gold discs inside silicon nitride pores. The gold discs were ordered in arrays of various diameters (24–60 nm) and different pitches (0.25–2 μm). The patterned samples were then transferred to the growth chamber of the metal–organic molecular beam epitaxy (MOMBE) system [34]. Trimethylindium (TMI) served as the precursor for indium. Thermally (pre-)cracked phosphine (PH_3_) and arsine (AsH_3_) served as group-V precursors. Prior to growth, the substrate was heated to the growth temperature under 3 SCCM of phosphine flow. As shown in Figure 1a, in this growth system, the metal–organic precursor beam is perpendicular to the sample, while the group-V beam is 30° inclined. Figure 1b shows a schematic of asymmetric core–shell growth leading to the bending, brought by the inclined group-V molecular beam inlet.

InP NW were grown for 35 min at 420 °C by introducing TMI flux corresponding to a growth rate of 0.11 micron per hour at 500 °C on (100) oriented substrates. Afterward, growth was switched between InAs and InP. This was done through 4 periodic interchanges of InAs and InP, 50 s for each segment, by changing group-V precursor from PH_3_ (3 SCCM) to AsH_3_ (2 SCCM). Two types of gas-switching schemes were used: (i) with TMI interruption during switching (type A) and (ii) without TMI interruption during switching (type B). Each growth sequence was applied to a different sample. Nanowire characterization was done using high-resolution scanning electron microscopy (HRSEM, Hitachi 4700, Tokyo, Japan). Transmission electron microscopy (TEM) was carried out by Titan 80-300 FEG-S/TEM (FEI, Waltham, MA, USA) instruments. The TEM samples were prepared by scratching nanowires from the samples onto a carbon grid. COMSOL 5.3a was used for finite element simulations.

## 3. Results and Discussion

**Figure 2:**
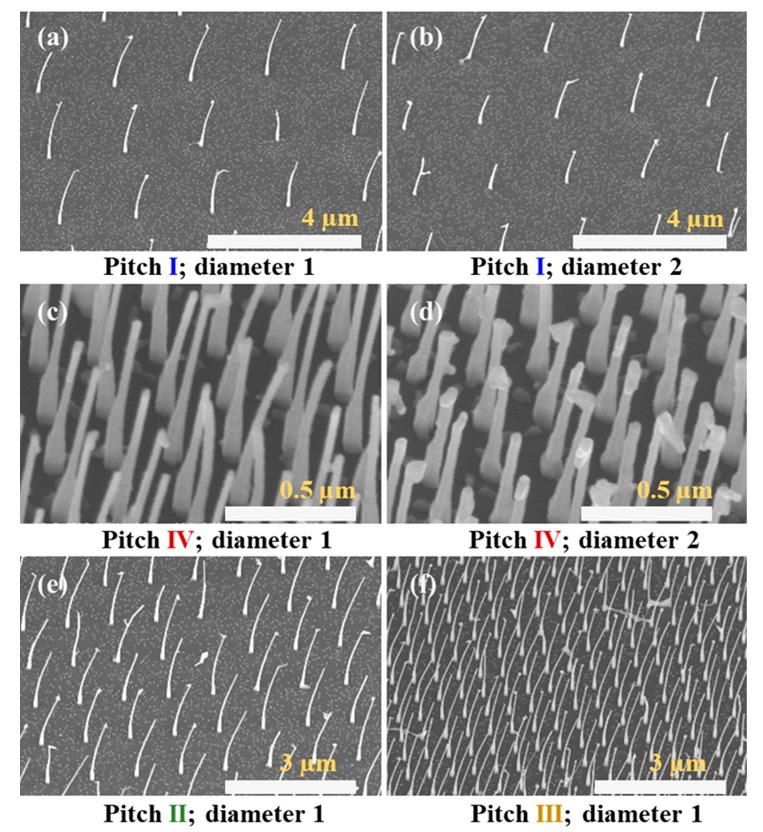
SEM images of bent heterostructure grown from catalysts of various pitch and diameter, with diameter 1 (~38 nm) and diameter 2 (~43 nm), and wire pitch of (**a**,**b**) 2 µm, (**c**,**d**) 0.25 µm, (**e**) 1 µm, and (**f**) 0.5 µm. The main finding was the reduction of bending with increasing NW density and diameter.

Figure 2 shows SEM images of the growth results from the first switching sequence examined with different pitches and diameters (correlated to the e-beam dose used). The average wire length for the 0.5 µm pitch was 1850 ± 100 nm for type A and 2200 ± 180 nm for type B. We will not discuss the effects of the switching sequence further as it was not found to deeply affect NW bending.

**Figure 3:**
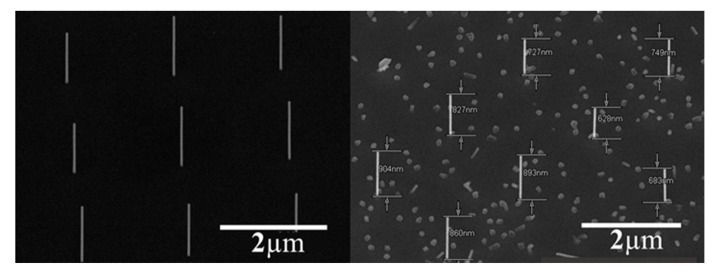
Homoepitaxial growth of III–V NWs. Left hand side, InP NWs on InP <111> (following [38]) and right hand side, InAs. This type of growth did not result in any bending or radial asymmetry, demonstrating that bending and asymmetry arise due to heterostructure growth.

We made several observations: (i) the NWs were already bent on the growth substrate, all to the same direction; (ii) bending diminished with decreasing pitch; and (iii) bending diminished with increasing diameter. The first observation implies an inherent asymmetry brought by the heterostructure growth, probably due to the asymmetric growth chamber. As mentioned earlier, the directionality of MBE allows control over the bending direction [2]. The diminishing curvature with decreasing pitch can be related to shadowing effects during this growth [32,35]. In particular, a smaller pitch results in diminished NW growth rate, thus rendering the InP core stiffer (a cantilever bending force constant is proportional to *1*/*L*^3^, where *L* is length). Similarly, NWs of larger diameter are inherently stiffer and, in addition, grow slower than thinner NWs, thus contributing to reduced bending. In a recent publication, growth of InP–GaP core–shell NWs in our system was reported [36]. In that case, the group-III source was switched, and the group-V flow was maintained. Interestingly, this structure showed no bending despite nonuniform (though to a lesser extent than in this report) GaP shell growth. This was attributed to a thick InP core, which prevented bending, in agreement with the current observation. Furthermore, it is possible that scattering of arsenic growth species from the SiNx surface or adjacent NW sidewalls [37] (closer and densely packed in low pitch) results in averaging out the effect of directional impingement brought by growth chamber asymmetry. Also, bending was not related to the growth of any specific binary material as both InP and InAs NW did not show any bending (Figure 3). Previous studies of homoepitaxial InP NWs grown in this system have not shown cross-sectional asymmetry [33,38,39] unless deliberately induced [6].

**Figure 4:**
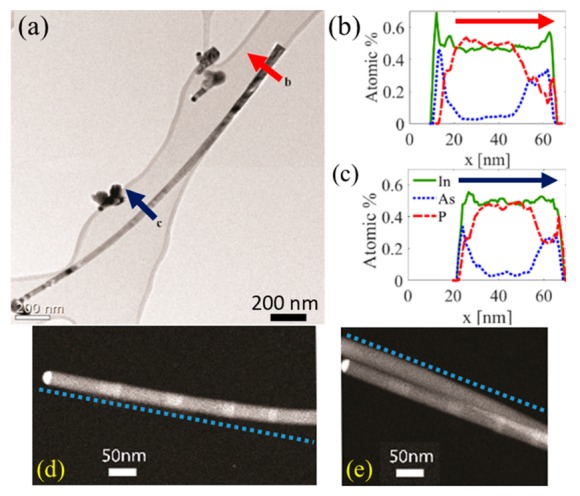
(**a**) TEM image of a bent InP–InAs heterostructure NW. (**b**,**c**) Energy-dispersive X-ray (EDX) analysis of the wire cross section at (**b**) wire base and (**c**) wire middle. Arrows show scan direction. (**d**,**e**) High-resolution TEM (HRTEM) scans of NW heterostructures showing further bending in addition to the creation of axial InAs segments. Dashed lines are a guide to the eye.

Following Lewis et al. [19], we expected to find material composition asymmetry along the NW cross section. Figure 4a shows a TEM image of a bent NW and corresponding energy-dispersive X-ray (EDX) composition scans at the NW base and middle (Figure 4b,c). Additional high-resolution TEM (HRTEM) scans of NWs (Figure 4d,e) showed bending at the NW middle/base and axial heterostructures at the top. The EDX scans showed composition asymmetry in the shell surrounding the InP core. From the measurements, it was unclear whether the shell was composed of alternating InP and InAs layers or an InAsP layer. Nonetheless, considering that switching group-V in MOMBE (chemical beam epitaxy; CBE) is generally fast [40,41], it is reasonable to believe that this was a low spatial resolution measurement of alternating layers. 

We assumed that the grown shell took the crystal structure of the core and that the shell was similarly strained by the core [12,36,42]. In our case, InP had a wurtzite (WZ) structure [33], and a WZ InAs shell will be strained at −3.13% (compressed, as the lattice constant of WZ InP in the c-axis is 0.6777 nm compared to 0.6996 nm in WZ InAs) [43]. Figure 4b,c indicates that the thicker part of the shell corresponded to the inner part of the bent NW. 

We moved on to a more quantitative analysis of the problem, establishing whether lattice mismatch was indeed the main contributor to NW bending. We carried out several COMSOL simulations with an input of NW shape, core–shell ratios, and a pre-existing strain in the shell. The resulting deformed NW curvature was compared to that of the NW shown in Figure 4. The experimental curvature radius was found manually (using an image processing software) to be ~4.3 µm. For a given curve, f(x), the radius of curvature is given by

(1) Rcurv(x)=(1+[f′(x)]2)32|f″(x)|

For nontapered NWs, we used two nonconcentric cylinders to form the core and the shell, while for tapered NWs, we used two truncated cones with a ratio of 0.6 between the top and bottom surface radii. Eight nontapered variants were considered, altering shell composition between InAs and InAs_0.5_P_0.5_ (to consider uncertainty in the composition), the fixed mechanical constraint (bottom surface or a single point on the bottom surface, see arrow on Figure 4a), and the shell dimensions between the thicker shell of Figure 4b and the thinner shell of Figure 4c. Two tapered variants were simulated using InAs as shell and altering between fixed point and fixed surface boundary conditions. The strain mismatch of InAs/InP, ε = −3.1% (−1.55% for InAsP) [43], was used as input for the simulation. The elastic properties of WZ InP and InAs were calculated following [44]: Young’s moduli in [0001] directions (Y_InAs_ = 101.73, Y_InP_ = 120.63, and Y_InAsP_ = 111.18 GPa), and the values for the stiffness matrix used for material properties in the numerical calculation. 

**Figure 5:**
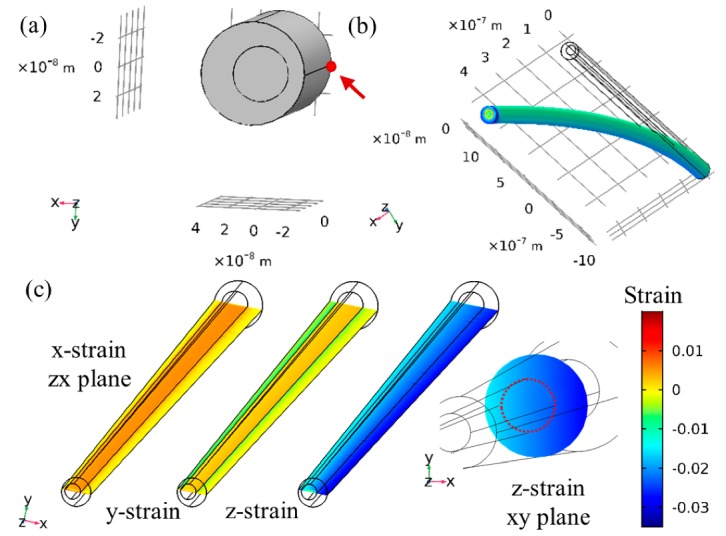
COMSOL simulation of the asymmetric shell-induced bending. (**a**) Top view of the geometry; (**b**) relaxed (bent) NW, where displacement of the top was nearly 0.5 µm. The red point and arrow mark the clamping in the point-clamped case; (**c**) strain analysis of the simulated results, i.e., the surface-clamped tapered case (grey tinted row in Table 1). The left-hand side shows x,y,z strains throughout the central ZX cross section, and the right-hand side shows the axial (z) strain throughout an XY cross section, with a dashed circle guiding the eye to the InP core. Overall, the NW was axially compressed and slightly strained radially following Poisson’s ratio. The scale bar is common to all cross sections.

The starting point of the simulation was a vertical NW, where the shell was precompressed by the lattice mismatch. Figure 5a shows a top view of the asymmetric core–shell structure. Elastic energy was released by bending of the structure toward the thicker shell (Figure 5b) in a strain sharing process, in agreement with Lewis et al. [19]. The NW profile at the thin shell was extracted and fitted to a parabola, and the radius of curvature was calculated at each point and on average using Equation (1). The results were compared to the results obtained manually for Figure 4 (~4.3 µm) and are given in Table 1. Two clear trends arose from the simulation: (i) bending increased with the thicker shell due to increase in the effect of the lattice mismatch; (ii) bending increased with lattice mismatch (InAs compared to InAsP), indicating the prominent role of the initial strain in the system. The simulations of nontapered NW underestimated the experimental bending of the NW; however, the tapered NW geometry presented a remarkable agreement with the experimental finding.

The successful simulation of the experimental curvature indicated that strain relaxation was the dominant factor in the bending of the heterostructure NWs and that an ability to control growth would allow strain engineering within the NW. Note that thermal relaxation was not a likely mechanism as the differences in thermal expansion coefficients of InP and InAs would correspond to a strain <0.1% [45,46], which is negligible compared to the lattice mismatch strain. 

There are several implications of such results. In particular, we consider the resulting strains in the simulation which provided the best fit to the experimental result, i.e., the tapered NW (we focused on the surface clamped case). Figure 5c shows the three components of strain in the central ZX plane and the z-axis (axial) component in a mid-wire XY plane. The most general result was that the InAs shell induced axial compression in the entire NW and a corresponding expansion in the radial directions, mostly in the x component of the InP core. This would be expected due to y-axis symmetry. Therefore, the addition of the relatively thin shell resulted in about 2% axial compression in the core, which was fairly uniform throughout the plane. One can think of an emitter embedded within the NW, now experiencing 2% compression, which is expected to significantly change its optical emittance [47], as has been demonstrated by Lewis et al. [19]. Furthermore, in WZ structures, and GaN technology in particular, this could be used to compensate for inherent electrical fields arising from spontaneous polarization [48]. The conductivity of the core or the shell could be controlled through the strain. For example, the conductance of an InAs NW was reported to change by 2 orders of magnitude through a compression of 2% through surface state modification [29]. This could have dramatic effects on the electronic properties of a compressed InP/InAs heterostructure, such as ours. 

## 4. Summary

We have demonstrated the growth of asymmetric InP/InAs core/shell NWs. The NWs presented a bent geometry throughout the sample in the same direction. Specifically, the NWs bent inwards toward the thicker part of the InAs shell. We attribute this result to the growth process, which was carried out without rotation in an MOMBE system where the group-V inlet is inclined compared to the substrate normal. Finite element simulation confirmed the dominance of lattice mismatch and subsequent strain sharing in NW bending. Simulation results for NW curvature based on a particular NW geometry and composition were in remarkable agreement with the experiment (~10%). The correspondence between simulated composition/dimensions and resulting geometry and experimental geometry shows that control of NW growth can serve as an effective and reliable design tool for strain and shape engineering. A significant compression of the entire NW was found in the range of 2%. Such compression/strain could be applied as a route to tune light emission from nanowires or their conductivity and electronic states.

## Figures and Tables

**Table 1:** COMSOL results for the relaxed radius of curvature.

Shell Composition	Shell Thickness (Thick; Thin) [nm]	Clamping	Radius of Curvature [µm]
InAs	11; 5.5	Surface	6 ± 0.4
InAs	8; 2	Surface	7.5 ± 0.31
InAsP	11; 5.5	Surface	11.6 ± 0.2
InAsP	8; 2	Surface	14.7 ± 0.16
InAs	11; 5.5	Point	7.2 ± 0.08
InAs	8; 2	Point	9 ± 0.07
InAsP	11; 5.5	Point	13.4 ± 0.04
InAsP	8; 2	Point	17 ± 0.04
InAs	Tapered	Surface	3.7 ± 0.55
InAs	Tapered	Point	4.8 ± 0.12
